# Insights about the structure of farnesyl diphosphate synthase (FPPS) and the activity of bisphosphonates on the proliferation and ultrastructure of *Leishmania* and *Giardia*

**DOI:** 10.1186/s13071-020-04019-z

**Published:** 2020-04-05

**Authors:** Ana Paula R. Gadelha, Claudia Maia Brigagao, Martha Barros da Silva, Aline Beatriz Mello Rodrigues, Ana Carolina Ramos Guimarães, Fernando Paiva, Wanderley de Souza, Cristina Henriques

**Affiliations:** 1 Laboratório de Biotecnologia, Instituto Nacional de Metrologia (INMETRO), Rio de Janeiro, RJ Brazil; 2 Universidade do Grande Rio -UNIGRANRIO, Rua Prof. José de Souza Herdy, 1.160 - Jardim 25 de Agosto, Duque de Caxias, RJ Brazil; 3 Laboratório de Ultraestrutura Celular Hertha Meyer (LUCHM, IBCCF-UFRJ), Universidade Federal do Rio de Janeiro, Ilha do Fundão, Rio de Janeiro, RJ Brazil; 4 Laboratório de Genômica Funcional e Bioinformática, Instituto Oswaldo Cruz (IOC, Fiocruz), Manguinhos, Rio de Janeiro, RJ Brazil; 5 Departamento de Patologia, Centro de Ciências Biológicas e da Saúde da UFMS, Universidade Federal do Mato Grosso do Sul, Cidade Universitária, Campo Grande, MS Brazil; 6 Fiocruz Mato Grosso do Sul, Rua Gabriel Abrão, 92 Jardim das Nações, Campo Grande, MS Brazil; 7 Laboratório de Biotecnologia e Fisiologia de Infecções Virais-LABIFIV, Instituto Oswaldo Cruz (IOC, Fiocruz), Av Brasil 4365, Manguinhos, Rio de Janeiro, RJ Brazil

**Keywords:** Bisphosphonates, Farnesyl diphosphate synthase, FPPS, *Leishmania*, *Giardia*, Protozoan, Isoprenoid, Isoprenylation, Sterol, Ergosterol

## Abstract

**Background:**

The enzyme farnesyl diphosphate synthase (FPPS) is positioned in the intersection of different sterol biosynthesis pathways such as those producing isoprenoids, dolichols and ergosterol. FPPS is ubiquitous in eukaryotes and is inhibited by nitrogen-containing bisphosphonates (N-BP). N-BP activity and the mechanisms of cell death as well as damage to the ultrastructure due to N-BP has not yet been investigated in *Leishmania infantum* and *Giardia*. Thus, we evaluated the effect of N-BP on cell viability and ultrastructure and then performed structural modelling and phylogenetic analysis on the FPPS enzymes of *Leishmania* and *Giardia*.

**Methods:**

We performed multiple sequence alignment with MAFFT, phylogenetic analysis with MEGA7, and 3D structural modelling for FPPS with Modeller 9.18 and on I-Tasser server. We performed concentration curves with N-BP in *Leishmania* promastigotes and *Giardia* trophozoites to estimate the IC_50_*via* the MTS/PMS viability method. The ultrastructure was evaluated by transmission electron microscopy, and the mechanism of cell death by flow cytometry.

**Results:**

The nitrogen-containing bisphosphonate risedronate had stronger anti-proliferative activity in *Leishmania* compared to other N-BPs with an IC_50_ of 13.8 µM, followed by ibandronate and alendronate with IC_50_ values of 85.1 µM and 112.2 µM, respectively. The effect of N-BPs was much lower on trophozoites of *Giardia* than *Leishmania* (IC_50_ of 311 µM for risedronate). *Giardia* treated with N-BP displayed concentric membranes around the nucleus and nuclear pyknosis. *Leishmania* had mitochondrial swelling, myelin figures, double membranes, and plasma membrane blebbing. The same population labelled with annexin-V and 7-AAD had a loss of membrane potential (TMRE), indicative of apoptosis. Multiple sequence alignments and structural alignments of FPPS proteins showed that *Giardia* and *Leishmania* FPPS display low amino acid identity but possess the conserved aspartate-rich motifs.

**Conclusions:**

*Giardia* and *Leishmania* FPPS enzymes are phylogenetically distant but display conserved protein signatures. The N-BPs effect on FPPS was more pronounced in *Leishmania* than *Giardia*. This might be due to general differences in metabolism and differences in the FPPS catalytic site.
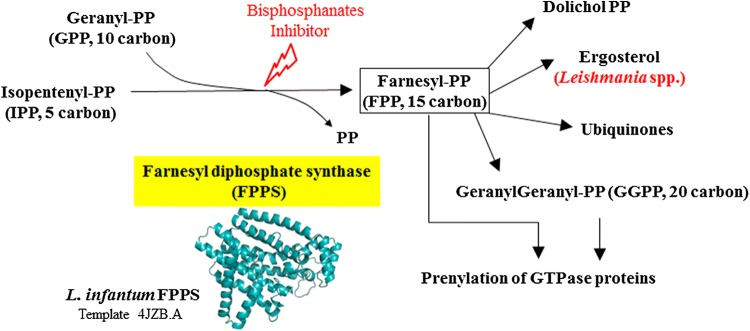

## Background

Farnesyl diphosphate synthase (FPPS) is a key enzyme in sterol metabolism. It is positioned at the intersection of different pathways, including those involved in the biosynthesis of isoprenoids, dolichols, ubiquinones and ergosterol/cholesterol. *Giardia* and other early diverging eukaryotes do not synthesize ergosterol or cholesterol *de novo* in contrast to *Leishmania* and trypanosomatids that synthesize ergosterol instead of cholesterol, which is produced by humans and other mammals.

The pathway for ergosterol biosynthesis includes enzymes that differ from cholesterol biosynthesis, making the ergosterol biosynthesis pathway a potential target for chemotherapy [1, 2]. Other pathways and enzymes of sterol metabolism include isoprenoid/prenylation and the dolichol biosynthesis. These pathways are ubiquitous in eukaryotes but have not received much attention. Genomic analysis has facilitated prediction of several metabolic pathways among eukaryotic organisms [3] and these predicted pathways enable comparisons to be made between sterol metabolism in early branching protozoans such as *Giardia* and *Leishmania*.

Leishmaniasis is a complex of diseases. There are more than 20 *Leishmania* species that cause different diseases, i.e. visceral leishmaniasis (VL), cutaneous leishmaniasis (CL) and mucocutaneous leishmaniasis (MCL). Leishmaniasis occurs in 102 countries, and CL is the most common and widespread [4]. More than 70% of the CL cases occur in 10 countries: Afghanistan, Algeria, Brazil, Colombia, Costa Rica, Ethiopia, the Islamic Republic of Iran, Peru, Sudan and the Syrian Arab Republic [4]. Around 90% of the global VL cases are reported in only six countries: Bangladesh, Brazil, Ethiopia, India, South Sudan and Sudan. In the Americas, *Leishmania infantum* is the etiological agent of VL [5], which is lethal if not treated. Brazil has a high burden of CL and VL with an incidence rate of 1.46 and 0.41 cases per 10,000 inhabitants, respectively [4, 6], CL cases are widespread throughout the Brazilian national territory and VL cases are reported in 21 states [7]. Leishmaniasis has been spread to previously non-endemic areas including urban centers. Indeed, nearly 1600 Brazilian cities have autochthonous transmission [7].

*Giardia* is the causative agent of giardiasis. It is a major cause of diarrhea in humans and an important public health problem [8, 9]. *Giardia duodenalis* (syn*. G. intestinalis* and *G. lamblia*) is divided into eight genetic assemblages (A-H) [10, 11] and possesses two morphological forms: trophozoites that infect the duodenum; and cysts that facilitate disease transmission by contaminating soil, food, and water following excretion in the feces. *Giardia duodenalis* assemblages A and B are responsible for human giardiasis and these types are globally distributed [9, 10, 12].

*Giardia* sterol metabolism is restricted to a few metabolic pathways [13] including the isoprenoid, the dolichol, and the ubiquinone or coenzyme Q (CoQ) pathways. CoQ is a component of the electron transport chain in aerobic organisms such as *Leishmania*, but is detected at much lower levels in *Giardia*, which has a poorly developed endomembrane system and lacks organelles including the Golgi and mitochondria [14, 15].

In contrast to *Giardia*, *Leishmania* has a complex life-cycle and sterol metabolism. It has adapted to a life-cycle that alternates between the promastigote (the infective form found inside the phlebotomine vector) and the amastigote form that resides inside the macrophages of the mammalian host. *Leishmania* has a sophisticated endo-membrane system, evolved mitochondria, and possesses the main enzymes and pathways of sterol metabolism. The enzyme profile of sterol metabolism and the presence of sterol-metabolizing gene sequences in the genome of *Giardia* and *Leishmania* suggest that the five carbon isoprene units, isopentenyl diphosphate (IPP) and its isomer dimethylallyl diphosphate (DMAPP), are synthesized *via* the mevalonate pathway (MEV) [3]. The IPP and DMAPP metabolites are substrates of farnesyl diphosphate synthase (FPPS) and lead to production of 15 carbon farnesyl diphosphate (FPP).

FPP is a key intermediate of sterol metabolism with a role in the post-translational modification of proteins *via* farnesyl transferase as well as in protein prenylation of the Ras superfamily of small GTP-binding proteins. FPP is also the precursor of several biomolecules with distinct biological function including the polyisoprenoids composed of 11 to 23 isoprene units known as dolichols [16].

Dolichols are carriers of N-glycan and glycosylphosphatidylinositol (GPI). They are inserted in the internal membrane of the endoplasmic reticulum (ER) and have a role in post-translational modification of proteins. *Leishmania* and *Giardia* produce dolichols with 11 to 12 isoprene units [17, 18].

*Giardia* lost the capacity to synthesize ergosterol and cholesterol *de novo* during evolution, but it does possess the enzymes of the MEV pathway including FPPS. Comparative analyses based on profiling of sterol biosynthethic enzymes of 46 eukaryotic proteomes showed that farnesyl/geranyl diphosphate synthase (FPPS and GPPS) and farnesyl transferase complex are ubiquitous in all organisms studied, including *Giardia*. This indicates that isoprenoid production is indispensable for all eukaryotes [3]. *Giardia* FPPS displays the conserved motifs and protein signatures found in FPPS of other organisms but has low identity with FPPS of humans, and *Leishmania* as evaluated previously by multiple sequence alignment and phylogenetic analyses [19]. In *L. major*, the FPPS structure was elucidated *via* crystallography [20].

Functional characterization of the recombinant FPPS has demonstrated that the enzyme is strongly inhibited by nitrogen-containing bisphosphonates (N-BP) such as risedronate [21, 22]. N-BPs have been the frontline treatment for bone disorders including osteoporosis, tumor-associated bone disease, and Paget’s disease [23]. N-BPs lead to depletion of FPP and GGPP isoprenoids and required prenylation of small GTPase proteins. The failure of protein prenylation due to N-BP is one of the main mechanisms behind decreased bone resorption by osteoclasts [24]. Bisphosphonates are also shown to be active against some protozoans [19, 25] but have not been tested on *L. infantum* and *G. duodenalis*. Furthermore, the mechanism of death and the effect on mitochondrial function and ultrastructure due to N-BP treatment has not been rigorously explored in parasitic protozoans.

We performed molecular modelling of FPPS sequences from *L. infantum* and from the distantly related FPPS enzyme of *Giardia.* Phylogenetic analysis of *Leishmania* FPPS and of different isolates of *Giardia* was also performed. We tested the effect of N-BPs on the protozoan *L. infantum* and *Giardia* to evaluate its effects on protozoan proliferation, viability and ultrastructure. Our results suggest that the isoprenoid pathway may represent an interesting target for evaluating mechanisms of cell death and a target for anti-parasitic drugs.

## Methods

### Parasite culture

Promastigotes of *Leishmania infantum* MHOM/BR/74/PP75 (IOCL0579, CLIOC—http://clioc.fiocruz.br) were grown in Schneider’s medium (Sigma-Aldrich, São Paulo, Brazil) supplemented with 10% fetal calf serum (FCS) maintained at 26 °C until the logarithmic growth stage. Trophozoites of *G. duodenalis* WB strain (clone C6; ATCC No. 30957) were cultivated in TYI-S-33 medium [26], pH 7.2, supplemented with 0.1% bovine bile and 10% FCS. Cultures were maintained at 37 °C.

### Multiple sequence alignment and phylogenetic reconstruction

A Basic Local Alignment Search Tool (BLASTp) search was performed with FPPS sequences of proteins experimentally characterized and deposited in the Protein Data Bank (PDB) and UniProtKB/Swiss-Prot (Table 1).

**Table 1:** FPPS protein sequences

Organism	Accession number	References
*Homo sapiens*	P14324.4^a^; 4LFV^b^	[39]
*Saccharomyces cerevisiae*	P08524.2^a^	[50]
*Leishmania major*	Q4QBL1^a^; 4JZX^b^; 4JZB^b^, 4K10^b^; XP_001683287.1^c^	[20]
*Trypanosoma cruzi*	EKG07068.1^e^; 1YHK^b^; 1YHL^b^; 1YHM^b^	[51]
*Trypanosoma brucei brucei* TREU927	Q57WF1^a^; 2EWG^b^; XP_845959.1^c^	[52]
*Escherichia coli* (strain K12)	P22939^a^; 1RQI^b^	[53]
*Arabidopsis thaliana* (thale cress)	Q43315^a^; NP_193452.1^c^	[54]
*Leishmania donovani*	ABI16061.1^e^	
*Leishmania infantum* JPCM5	E9AH04^a^; XP_003392505.1^c^	
*Leishmania mexicana*	XP_003875590.1^c^	
*Leishmania braziliensis* MHOM/BR/75/M2904	A4HCH8^a^; XP_001565042.1^c^	
*Crithidia fasciculata* strain Cf-Cl	CFAC1_240021200.1-p1^d^	
*Giardia intestinalis* ATCC 50803	XP_001709477.1^c^; GL50803_6633-t26_1-p1^f^	
*Giardia intestinalis* ATCC 50581	EES99536.1^e^; GL50581_3281-t26_1-p1^f^	
*Giardia lamblia* P15	EFO63579.1^e^; GLP15_4726-t26_1-p1^f^	
*Shigella*	WP_000347239.1^c^	
*Enterobacteriaceae*	WP_000347220.1^c^	

^a^UniProtKB/Swiss-Prot database

^b^PDB database

^c^NCBI database

^d^TriTrypDB database

^e^GenBank database

^f^GiardiaDB database

The sequences were download in FASTA format to perform multi-alignment and phylogenetic analysis. Multiple sequence alignment was performed using MAFFT v7 (EMBL-EBI search and sequence analysis tool; https://www.ebi.ac.uk/Tools/msa/mafft/) applying the BLOSUM62 matrix 1.53 gap open penalty and default parameter settings [27]. Phylogenetic analysis of FPPS sequences was performed in MEGA 7 [28, 29]. The evolutionary history was inferred by the Maximum Likelihood method based on the JTT matrix-based model [30]. The bootstrap consensus tree inferred from 1000 replicates was used to represent the evolutionary history of the analyzed taxa [31]. Initial tree(s) for the heuristic search were obtained automatically by applying Neighbor-Join and BioNJ algorithms to a matrix of pairwise distances estimated using a JTT model. The topology with superior log likelihood values were then selected.

### Theoretical modeling of FPPS

The amino acid sequences of *Leishmania infantum* and *Giardia intestinalis* (Assemblage A isolate WB; gene: GL50803_6633) related to farnesyl pyrophosphate synthase were used to construct the 3D theoretical structure models of this enzyme. These sequences were subjected to BLASTp searches (https://blast.ncbi.nlm.nih.gov/Blast.cgi?PAGE=Proteins) to identify potential template structures from PDB for prediction of *Giardia* and *Leishmania* FPPS structures. Regarding the FPPS target sequence for *L. infantum*, 50 models were generated with the standard auto model routine and optimized *via* the variable target function method (VTFM) until 300 iterations were achieved using Modeller version 9.18 [32] and PDB 4JZB [20] as the template. The model with the lowest discrete optimized protein energy (DOPE) value was selected.

The system’s energy was minimized using Chimera software by applying the default parameters (http://www.rbvi.ucsf.edu/chimera). Additionally, the sequence of *Giardia* WB FPPS was submitted to the I-Tasser server (https://zhanglab.ccmb.med.umich.edu/I-TASSER/) to obtain a model *via* a threading prediction. Both models were submitted to the SAVES server (http://servicesn.mbi.ucla.edu/SAVES/) to be evaluated by validation programs (Ramachandran plot, ERRAT and Verify3D). The three-dimensional structures were generated using PyMOL [33]. All structures have electrostatic surface maps created using PyMOL with the APBS plugin (default parameters) predicted by assuming a pH 7.0.

### *In vitro* assays of bisphosphonate in *Leishmania* and *Giardia*

The N-BP and inhibitors of FPPS (Sigma-Aldrich) including alendronate sodium trihydrate (A4978), ibandronate sodium salt (I5784), neridronate (N6037), pamidronate disodium salt hydrate (P2371), and risedronate sodium (SML0650) were diluted in water to prepare stock solutions. Concentration curves were performed on *Leishmania* promastigotes at 10^6^ promastigotes/ml in Schneider’s medium supplemented with 10% FCS. Concentration curves were performed for each N-BP with increasing concentrations: 10, 20, 40, 80, 100, 200 and 400 µM. Promastigotes were incubated with each compound for 72 h at 26 °C.

*Giardia duodenalis* trophozoites were cultivated in Eppendorf tubes containing 10^5^ trophozoites/ml in TYI-S-33 medium supplemented with 10% FCS. Concentration curves were performed for each N-BP inhibitor with increasing concentrations: 10, 50, 100, 200, 500 and 1000 µM for 48 h at 37 °C.

The IC_50_ was estimated using the viability method using tetrazolium 3-(4,5-dimethyl-2-thiazolyl)-5-(3-carboxymethoxyphenyl)-2-(4-sulfophenyl)-2H (MTS) and 5-methyl-phenazinium methyl sulfate (PMS) [34]. Thereafter, the IC_50_ was estimated by non-linear regression using the Sigma plot software.

### Tetrazolium salt-based viability assay

Parasite cultures of *Leishmania* promastigotes and *G. duodenalis* trophozoites were centrifuged at 2000×*g* for 10 min at 4 °C. Before centrifugation, *G. duodenalis* trophozoites were placed on ice for 10 min and then shaken to detach the parasites. After centrifugation, the medium was removed and the pellet was suspended with the same volume of saline buffer composed of 21 mM HEPES, 0.7 mM Na_2_PO_4_, 137 mM NaCl, 5 mM KCl at pH 7.4 supplemented with 6 mM glucose (SBG). For each condition, 100 µl of each parasite cell suspension was transferred in triplicate to a 96-well plate. To perform the negative control, 100 µl of each parasite suspension was transferred in duplicate and fixed with 0.4% paraformaldehyde. Afterwards, 20 µl of MTS/PMS mixture was added to each well containing 100 µl of protozoan in buffer SBG [34]. To produce the MTS/PMS mixture, 50 µl PMS (Sigma-Aldrich; P9625) stock solution was added to 1 ml of MTS stock solution (CellTiter 96 AQueous MTS Reagent Powder, G1112; Promega, São Paulo, Brazil).

### Electron microscopy

To evaluate the ultrastructure of protozoans treated with NB-Ps, *Leishmania* promastigotes and *G. duodenalis* trophozoites were incubated and fixed with 2.5% glutaraldehyde, 2.0% paraformaldehyde in cacodylate buffer, and post-fixed with 1% osmium tetroxide and 0.8% potassium ferrocyanide in 0.1 M cacodylate buffer (pH 7.4) for 1 h at room temperature. The samples were then washed, dehydrated in acetone, and embedded in Epon. Thin sections were stained with uranyl acetate and lead citrate and observed *via* transmission electron microscopy (Tecnai™ Spirit TEM; FEI Company, São Paulo, Brazil).

### Flow cytometry analysis of *L. infantum* promastigotes treated with alendronate

Programmed cell death was evaluated in *L. infantum* promastigotes treated for 24, 48 and 72 h with 100 µM alendronate and 10 µM miltefosine (M5571; Merck, São Paulo, Brazil) as a control. After treatment, parasites were analyzed by flow cytometry (BD Accuri™ C6; BD Biosciences, São Paulo, Brazil), and the BD Accuri C6 Software. Early and late apoptotic processes were distinguished using the vital dye 7-amino-actinomycin (7-AAD; BD Pharmingen, São Paulo, Brazil) as well as Annexin-V-FITC that binds to the exposed phospholipid phosphatidylserine (PS) in membranes (FITC Annexin V Apoptosis Detection Kit I; BD Pharmingen). Briefly, 1 ml of *L. infantum* culture with approximately 2–8 × 10^6^/ml promastigotes was centrifuged at 2000×*g* for 10 min, and the pellet was suspended in 100 µl of binding buffer according to the manufacturer’s suggestions. The 5 µl of annexin-V-FITC and/or 5 µl of 7-AAD (10 mg/ml) was added and incubated for 15 min. We then added 400 µl of binding buffer to a final volume of 500 µl. The samples were analyzed with a flow cytometer (BD Accuri C6, BD Biosciences), and 20,000 events were acquired. Controls were performed in promatigotes whose membranes had been permeabilized with 0.5% Triton X-100 for 15 min followed by incubation with 7-AAD (FL3) and/ or Annexin-V-FITC (FL1). The forward and side scatter plots (FSC-H × SSC-H) were used to evaluate the promastigote population with respect to cellular volume and shape.

The *L. infantum* promastigotes were incubated with 100 nM tetramethylrhodamine ethyl ester (TMRE, BD Pharmingen) to investigate the mitochondrial membrane potential after treatment with bisphosphonates. A stock solution of TMRE (1 mM) was prepared in DMSO and stored at − 20 °C. Thereafter, 1 ml of promastigotes incubated with bisphosphonates or miltefosine for 48 or 72 h, approximately 5–8 × 10^6^/ml, were centrifuged at 2000×*g* for 10 min. The pellets were suspended in 1 ml of saline buffer (137 mM NaCl, 5 mM KCl, 0.7 mM Na_2_HPO_4_, 6 mM glucose, and 21 mM HEPES, pH 7.3) and 0.1 µl of TMRE stock solution (FL2) was added and incubated for 15 min at room temperature. The promastigotes were then centrifuged, washed, and evaluated by flow cytometry (BD Accuri C6, BD Biosciences).

## Results

### *Giardia* and *Leishmania* FPPS sequence analysis

*Giardia* WB FPPS protein sequences display low identity to *Homo sapiens* (27.8%), *Saccharomyces cerevisiae* (29.9%), *L. infantum* (27.7%), *L. major* (28%) and *Escherichia coli* (20.9 %). FPPS sequences of *L. infantum* and *L. major* had a higher identity (~ 88%) to *Crithidia fasciculate*, than to *Trypanosoma cruzi* and *T. brucei* (~ 63%). The identity to higher-order eukaryotes was also lower; 43% for *Arabidopsis thaliana*, 37% for *Homo sapiens* and 34% for *S. cerevisiae*, with a coverage of 70–80%. The FPPS protein sequences of *Giardia* Assemblage A isolate WB displayed 97% identity to *Giardia* Assemblage B isolate GS, and 83% identity to *Giardia* Assemblage E isolate P15.

**Fig. 1:**
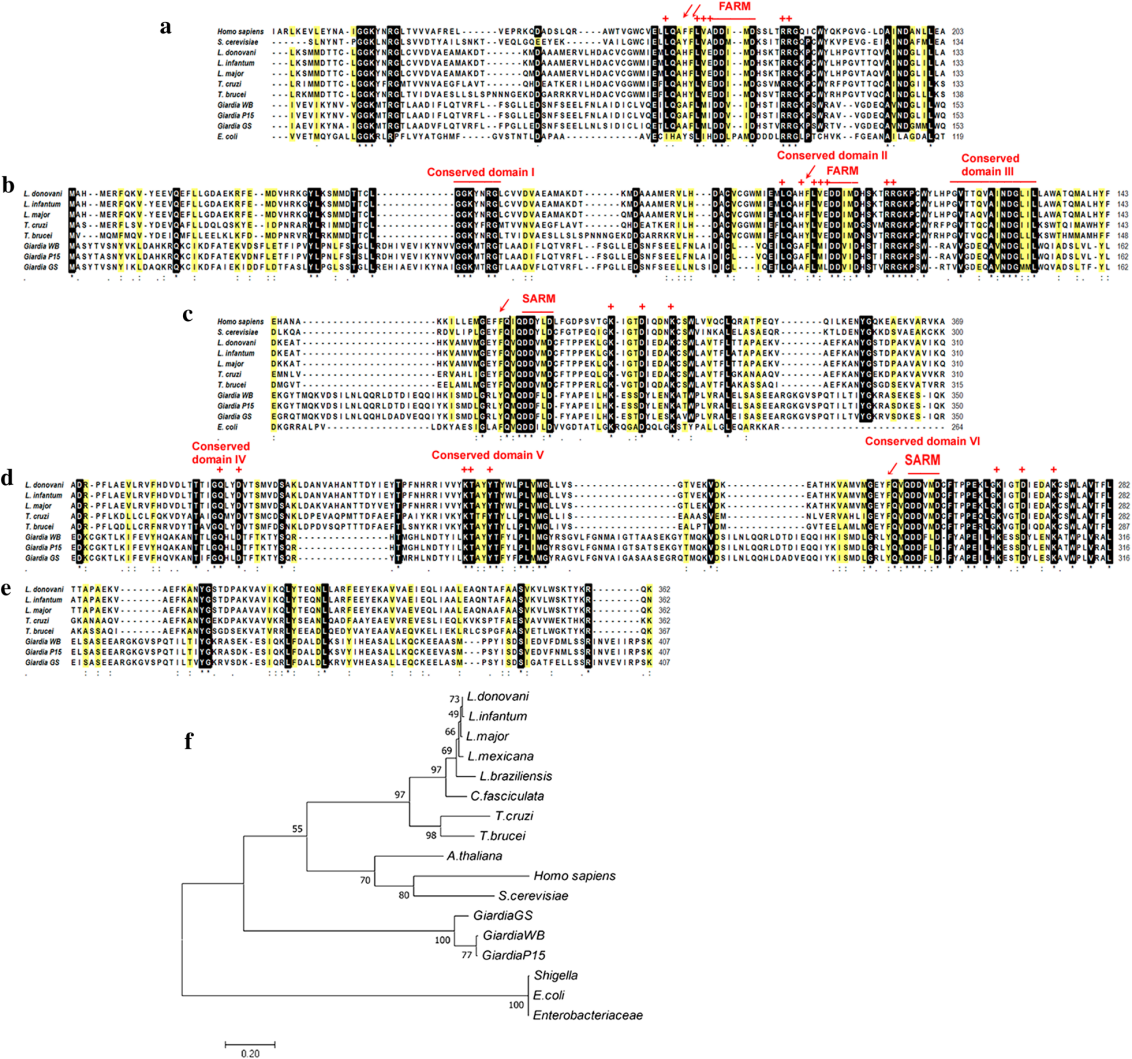
Evaluation of conserved motifs and phylogenetic analysis of *Giardia* and *Leishmania* FPPS sequences. Multiple sequence alignment by MAFFT v7.397 used the CLUSTAL format. First aspartate rich motif (FARM), marked with a red line: alignment of 11 organisms (**a**); alignment of trypanosomatid parasites and *Giardia* FPPS protein sequences (**b**). Second aspartate rich motif (SARM), highlighted with red line: alignment comparing 11 organisms (**c**); alignment of trypanosomatid parasites and *Giardia* FPPS protein sequences (**d**). C-terminal region of FPPS (**e**). Identical residues are highlighted in black, and similar amino acid residues are marked in yellow. Amino acids involved with binding sites (red cross); residues involved in the chain length of the product (red arrow). Phylogenetic tree, constructed with the maximum likelihood method (**f**)

Despite the low identity of *Giardia* FPPS with *Leishmania* and other eukaryotes, *Giardia* FPPS has conserved aspartate-rich motifs that are characteristic of FPPS enzymes. The first aspartate (D)-rich motif (FARM) is composed of DDXXD and is found in eukaryotic organisms (Fig. 1a, b). The second aspartate rich motif (SARM) has the DDXXD sequence (Fig. 1c, d). Crystallographic studies have shown that these motifs face each other and create a binding pocket. Both motifs are also involved in the catalytic site of the FPPS enzyme of several organisms, even in *E. coli*.

The FARM sequence motif is conserved from *Homo sapiens* to bacteria (Fig. 1a) and display two conserved arginine (R) residues and one lysine (K) downstream. These sequences are involved in binding of the substrate and coordination with Mg^+2^ ions (Fig. 1a). A comparison of the *L. major*, *L. infantum*, and *Giardia* WB FARM motif indicates that the conserved aspartate residues (bold) are **Asp**^98^(D), **Asp**^99^(D), Ile^100^(I), Met^101^(M) and **Asp**^102^(D) in *Leishmania*, and **Asp**^120^(D), **Asp**^121^(D), Val^122^(V), Ile^123^(I) and **Asp**^124^(D) in *Giardia* WB. Other conserved residues are two arginine units (bold): **Arg**^107^(R) and **Arg**^108^(R) in *Leishmania* and **Arg**^129^(R) and **Arg**^130^(R) in *Giardia* WB (Fig. 1b) as corroborated by InterPro (https://www.ebi.ac.uk/interpro/).

A comparison of the fifth and fourth amino acid residues before the FARM motif involved a chain length determination of the enzymatic product (GPP, FPP or GGPP). These are composed of His^93^(H), Phe^94^(F), Leu^95^(L), Val^96^(V) and Glu^97^(E) in *Leishmania* as well as Ala^115^(A), Phe^116^(F), Leu^117^(L), Met^118^(M) and Ile^119^(I) in *Giardia* (Fig. 1b). Phenylalanine (F) is a key residue involved in limiting the product chain length in *Leishmania*. The tyrosine (Y) residue has the same role in trypanosomes (Fig. 1b).

Multiple sequence alignment was performed to compare the SARM motif, which is composed of conserved aspartate residues in different organisms (Fig. 1c) and to compare the SARM motif and surrounding residues from trypanosomatids and *Giardia* (Fig. 1d). A sequence comparison between *Leishmania* and *Giardia* WB demonstrates the following conserved residues (bold) in *Leishmania*; Gln^167^(Q), **Asp**^170^(D), **Lys**^207^(K), Thr^208^(T), Tyr^211^(Y), **Asp**^250^(D), **Asp**^251^(D), **Asp**^254^(D) and **Asp**^268^(D). Similar residues were also found in *Giardia* such as Gln^187^(Q), **Asp**^190^(D), **Lys**^212^(K), Thr^213^(T), Tyr^216^(Y), **Asp**^285^D, **Asp**^286^D **Asp**^289^(D) and **Asp**^302^(D). The lysine (K) residues downstream of the SARM motif were **Lys**^264^(K) and **Lys**^273^(K) in *Leishmania* and **Lys**^298^(K) and **Lys**^307^(K) in *Giardia* (Fig. 1d). These lysines are also conserved in other organisms (Fig. 1c) and are involved in binding the substrate phosphate in coordination with Mg^+2^ [35]. Indeed, a phenylalanine (F) residue is observed upstream the SARM motif in all sequences of different organisms except for *Giardia* that has a lysine (K) residue (Fig. 1b). Previous work identified seven conserved regions in FPPS from trypanosomatids [19], the seventh region in the C-terminal region in *Giardia* has a conserved arginine residue and eight extra amino acids (Fig. 1e).

### Three-dimensional structure prediction and structural analyses

Here, the 3D model of the *L. infantum* FPPS was generated *via* comparative modeling based on the *L. major* structure (PDB ID 4JZB). The sequence alignment between *L. major* and *L. infantum* FPPS proteins had 96.95% identity with a coverage of 99%. We chose the model with the lowest DOPE scores (− 47931, 92188) among the 50 generated models. The Ramachandran plot of the selected model shows that 100% of all residues were allocated in energetically allowed regions with 99.2% in the favored region. The overall quality factor achieved with ERRAT was 92.6554, and the Verify 3D server estimated that 94.20% of the residues of *L. major* FPPS had an averaged 3D-1D score ≥ 0.2. These results indicate that the refined model has good quality and is reliable for further computational analysis.

The best results for the *Giardia* WB FPPS sequence were obtained with PDB 6B02 from a similarity search against PDB sequences using BLASTp [36]. The alignment between the *Giardia* WB FPPS and the template sequences showed that the identity and coverage were 29.57% and 81%, respectively. Structural prediction by threading methods was applied due to this low sequence identity with any other organism and the lack of experimentally elucidated three-dimensional structures. The Ramachandran plot of the resulting model had 93.8% of all residues allocated in energetically allowed regions with 76.2% in the favored region. The ERRAT analysis showed an overall quality factor higher than 81.7. Verify3D showed that 65.36% of the amino acid scored ≥ 0.2 in the 3D/1D profile. Thus, the *Giardia* FPPS theoretical model has sufficient quality to perform *in silico* analysis.

**Fig. 2:**
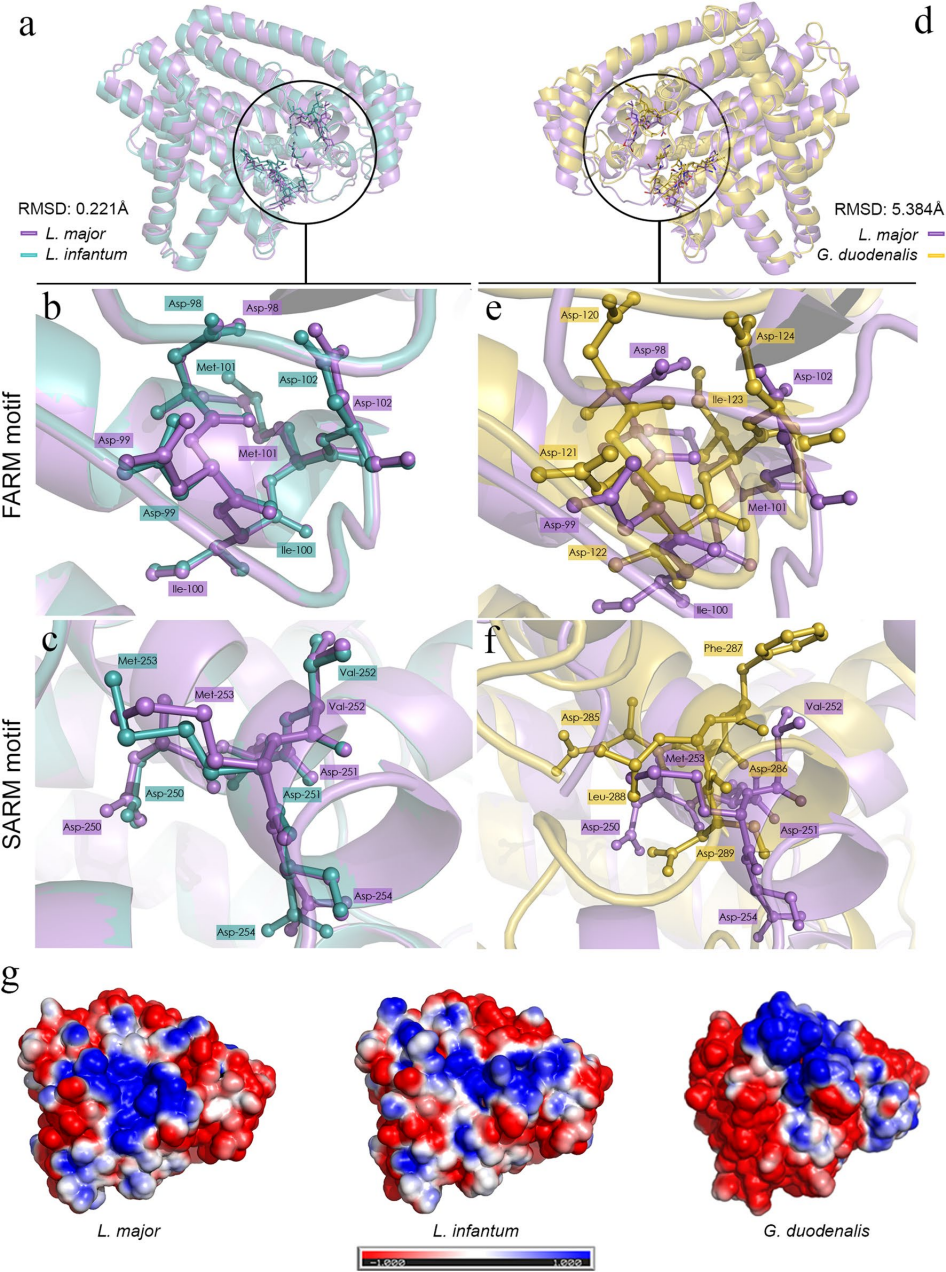
Structure comparison between FPPS of *Leishmania* and *Giardia*. Three-dimensional structure alignments based on the structural similarity of *L. major* FPPS crystallographic structure (PDB 4JZB, lilac chain A) with *L. infantum* (green) (**a**) and alignment of *L. major* FPPS (lilac) with *G. duodenalis* (yellow) (**d**). The inset highlights the residues (sticks) of FARM (**b**, **e**) and SARM (**c**, **f**) motifs in the catalytic site. Residues of both motifs, FARM and SARM, are conserved in genus *Leishmania* (**b**, **c**), and the residues of this region are not conserved between *L. major* and *G. duodenalis* (**e**, **f**). Electrostatic potential surface map generated by PyMol for the structures of FPPS (**g**). The color scale for surface electrostatic potential was set from − 1 kT/e (red) to 1 kT/e (blue). *Key*: white, neutral; blue, positive charge; red, negative charge

The structures are conserved when analyzing the alignment of the FPPS structures from organisms belonging to the *Leishmania* genus (Fig. 2a); the RMSD is 0.221 Å. The conserved residues found in FARM (DDIMD) and SARM (DDVMD) motifs for *L. major* and *L. infantum* are represented in Fig. 2b and c, respectively. However, the structural alignment between the *L. major* crystallographic structure and the *G. duodenalis* theoretical model displayed a lack of conserved residues that compose the regions outside the FARM and SARM motifs (Fig. 2d–f). The aspartic acid residues presented in FARM and SARM motifs (DDXXD) are part of the catalytic cavity and have an important role in protein function. Meanwhile, the residues represented by XX are the same for *L. major* and *L. infantum* in FARM (Fig. 2b) and SARM (Fig. 2c) but are different for *G. duodenalis* FARM (Fig. 2e) and SARM (Fig. 2f) motifs.

Importantly, even if there is a difference between the electrostatic potential on the surfaces of the three-dimensional structures of the FPPS, the site of the FARM and SARM motifs is electrostatically negative (Fig. 2g).

### Effect of N-BPs on *Leishmania* and *Giardia*

The expression of sterol biosynthetic enzymes are upregulated in the insect stage of *Trypanosoma brucei* and *Trypanosoma cruzi* procyclic and epimastigote forms [3]; thus, we evaluated N-BP inhibition on the promastigotes of *L. infantum*. *Giardia* has four enzymes of the MVA pathway: acetoacetyl-CoA thiolase, HMG-CoA-synthase, HMG-CoA reductase, and mevalonate kinase, expressed on the trophozoites stage [37].

We performed at least three assays with each bisphosphonate in the range of 5–500 μM on promastigotes of *L. infantum* for 72 h incubation. We also evaluated the cytotoxic effect of bisphosphonates on *G. duodenalis* trophozoites and created concentration curves from 10 μM to 1 mM for 48 h of incubation. Risedronate, ibandronate, and alendronate have increased antiproliferative activity on promastigotes of *L. infantum* (Table 2) *versus* trophozoites of *G. duodenalis* (Table 2). Only risedronate and ibandronate display antiproliferative activity (Table 2) in *G. duodenalis* as evaluated by the IC_50_.

**Table 2:** Effect of FPPS inhibitors on trophozoites of *Giardia duodenalis* and on promastigotes of *Leishmania infantum*

Nitrogen bisphosphonates (N-BP)	IC_50_ Mean ± SD (µM)	
	*L. infantum*	*G. duodenalis*
Risedronate	13.8 ± 6.0 (*n* = 5)	311 ± 120 (*n* = 3)
Ibandronate	85.1 ± 26.5 (*n* = 4)	271 ± 62 (*n* = 3)
Alendronate	112.2 ± 61.2 (*n* = 5)	nd
Pamidronate	118.7 ± 26.2 (*n* = 2)	nd
Neridronate	173.3 ± 31.6 (*n* = 3)	nd

*Note*: IC_50_ was estimated by the MTS method

### Effect of N-BPs on the ultrastructure of *Leishmania* and *Giardia*

**Fig. 3:**
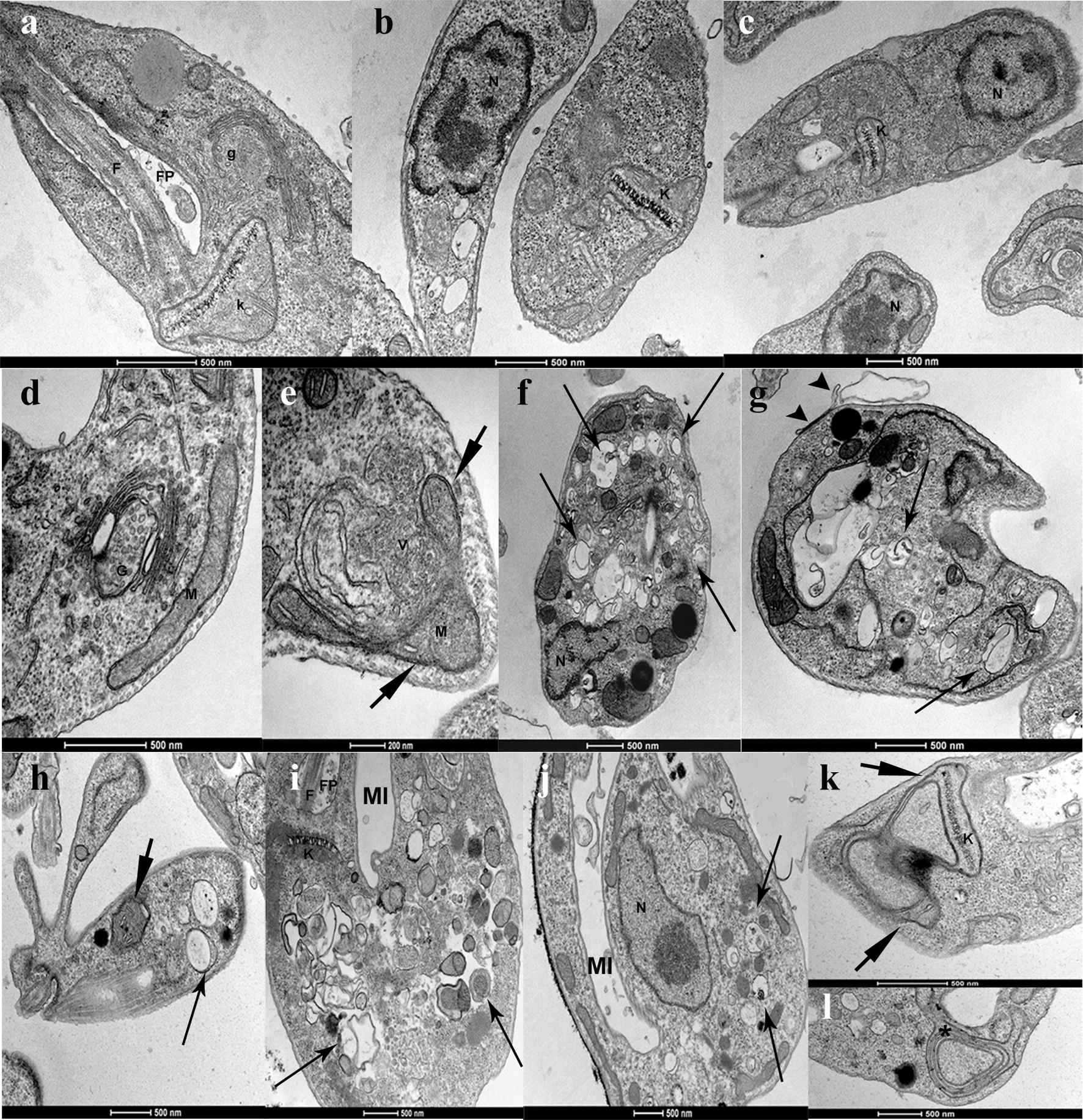
Ultrastructural evaluation of *L. infantum* treated with N-BP. Promastigotes of *L. infantum*: control, not treated (**a**–**c**); treated with risedronate (**d**–**g**); treated with alendronate (**h**–**l**). Mitochondrial swelling (short arrow); double membrane and vacuoles (long arrow); myelin figure (*); plasma membrane blebs (arrowhead). *Abbreviations*: N, nucleus; K, kinetoplast, M mitochondria; F flagellum; FP, flagellar pocket; G, Golgi; MI, membrane invagination. *Scale-bars*: **a**–**d**, 500 nm; **e**, 200 nm; **f**–**l**, 500 nm

*Leishmania* promastigotes treated with risedronate, ibandronate, and alendronate displayed the same ultrastructural alterations. To correlate the ultrastructural alterations caused by N-BPs with mechanisms of cell death in *Leishmania*, we evaluated the *L. infantum* promastigotes treated with 100 µM alendronate or 20 µM risedronate. Promastigotes accumulated small vesicles in the Golgi region near the kinetoplast (Fig. 3d, e) as well as mitochondrial swelling (Fig. 3e, k), altered cell division (Fig. 3h), formation of intracellular vesicles and lamellae (Fig. 3f-j), blebbing of the plasma membrane (Fig. 3g), as well as nuclear pyknosis and chromatin condensation (Fig. 3f, j). There was also an invagination of the plasma membrane in the flagellar pocket region without membrane rupture (Fig. 3i, j) as well as concentric membranes in regions of the mitochondria and myelin figures (Fig. 3k, l). Membrane integrity can distinguish apoptosis from necrosis. Promastigotes treated with alendronate have preserved plasma and nuclear membranes, as evaluated by electron microscopy.

**Fig. 4:**
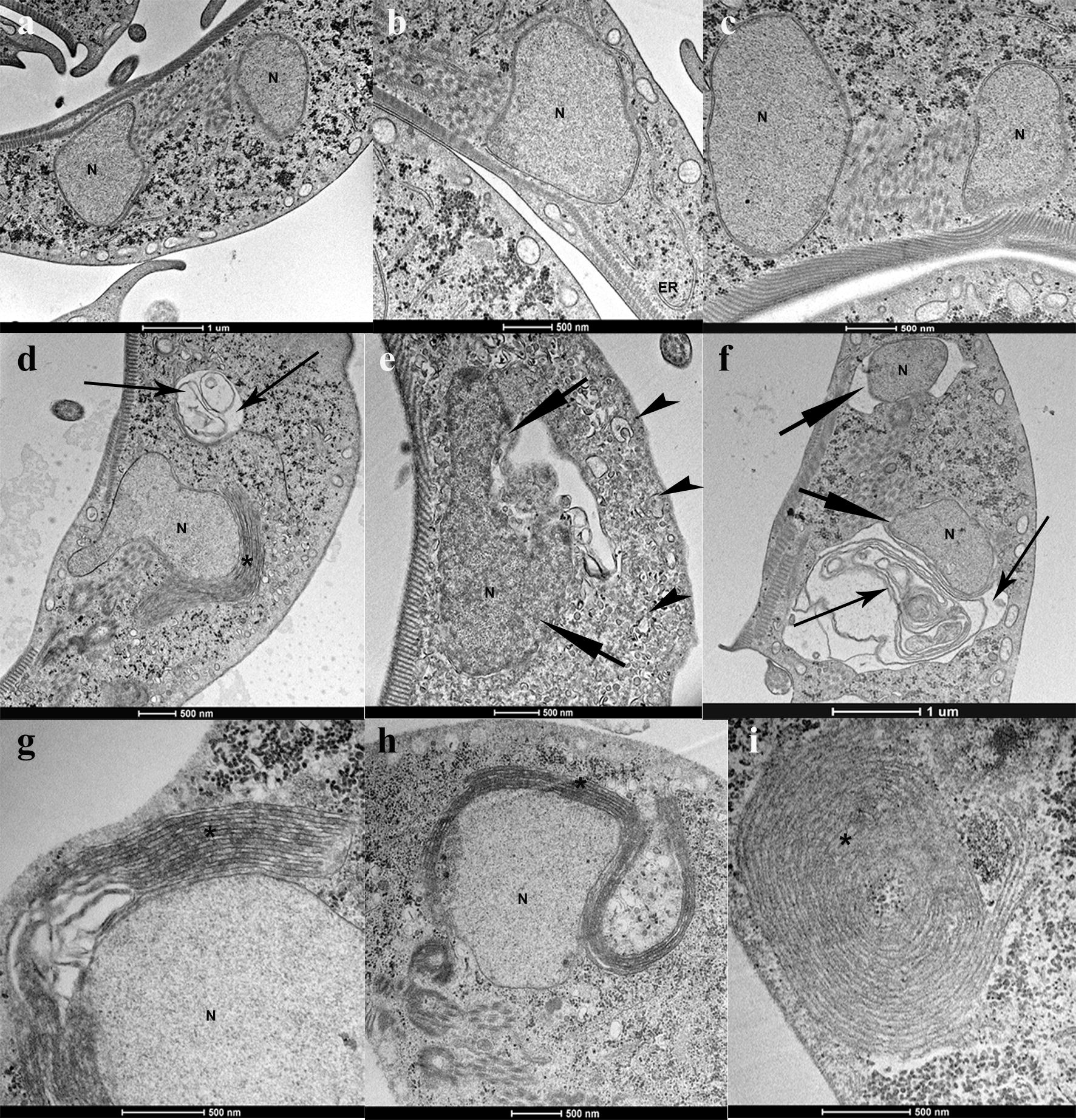
Ultrastructural evaluation of *Giardia* treated with N-BP. Trophozoites of *G. duodenalis*: control, not treated (**a**–**c**); treated with risedronate (**d**–**f**); treated with ibandronate (**g**–**i**). Nuclear pyknosis (short arrow); double membrane, intracellular vesicles, and lamellae (long arrow); small vesicles (arrowhead), myelin figure (*). *Abbreviations*: N, nucleus; ER, endoplasmic reticulum. *Scale-bars*: **a**, **f**, 1 µm; **b**–**e**, 500 nm; **g**–**i**, 500 nm

*Giardia duodenalis* treated with 300 µM risedronate or ibandronate for 48 h had a high frequency of concentric membranes near the nuclei as well as nuclear pyknosis and membrane layers and lamella formation on the nucleus (Fig. 4d, g, h). There was membrane detachment and formation of intracellular lamellae in the cytoplasm (Fig. 4d–f). Small vesicles were distributed in the cytoplasm (Fig. 4e). Intense myelin figures suggested nuclear engulfment (Fig. 4i), which can be caused by membrane accumulation from the endoplasmic reticulum. These ultrastructural alterations are due to disorganization of the endomembrane system.

### Evaluation of programmed cell death in *Leishmania* treated with N-BP

**Fig. 5:**
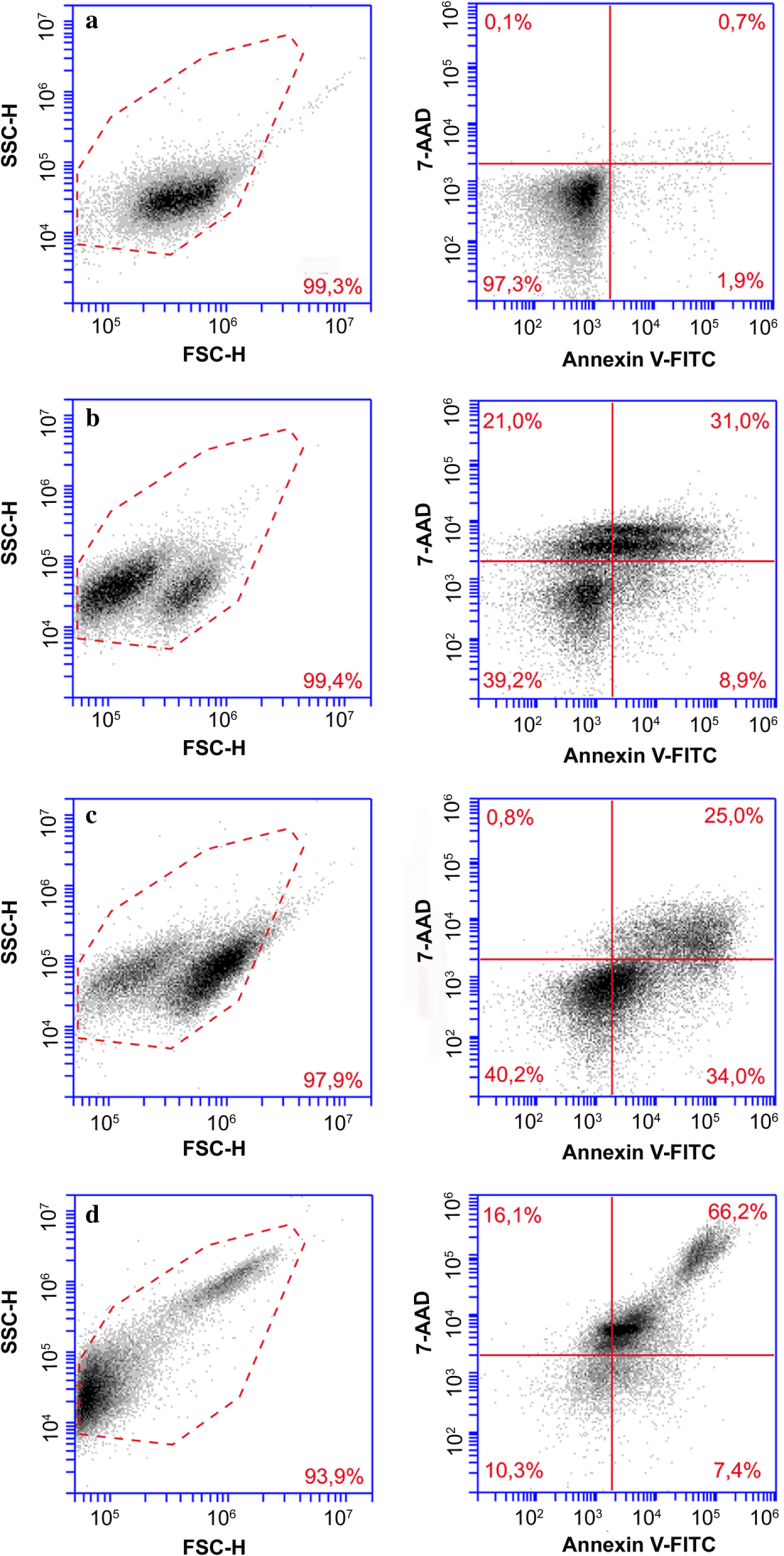
Analysis of cell death in promastigotes of *L. infantum* treated with alendronate. Negative control: promastigotes not treated with alendronate and stained with Annexin V and 7-AAD (**a**). Promastigotes treated with 10 µM miltefosine for 72 hours and stained with Annexin V and 7-AAD (**b**). Promastigotes treated with 100 µM alendronte for 72 hours and stained with Annexin V and 7-AAD (**c**). Promastigotes permeabilized with triton X-100, 0.5%, and stained with Annexin V and 7-AAD (**d**). SSC *versus* FSC (left panels); and FITC Annexin V (FL1) *versus* 7-AAD (FL3) (right panels)

We evaluated apoptosis and necrosis caused by N-BPs with three probes: (i) Annexin-V-FITC that labels exposed phosphatidylserine; (ii) 7-amino-actinomycin D (7-AAD) to evaluate plasma membrane integrity; and (iii) TMRE to estimate the loss of mitochondrial potential. A negative control comprised of viable promastigotes without N-BPs treatment was not stained with Annexin-V-FITC and/or 7-AAD. Control promastigotes after 72 h of cultivation did not undergo the process of apoptosis or necrosis (Fig. 5a).

The *L. infantum* promastigotes treated with 10 µM miltefosine for 48 or 72 h had two populations of promastigotes that increased with time as seen in the SSC × FSC plot (Fig. 5b). After 72 h of incubation with miltefosine, population 1 displayed co-staining with annexin-V and 7-AAD or just 7-AAD staining, and population 2 was not stained or displayed only annexin-V staining. Incubation of *L. infantum* promastigotes with 10 µM miltefosine induced co-staining in 12% and 31% after 48 and 72 h, respectively. Staining with only annexin-V was reported in 9.2% and 8.9 % and staining with 7-AAD was observed in 1.7% and 21% of promastigotes. These results indicate that miltefosine caused apoptosis and necrosis.

Promastigotes treated with 100 µM alendronate for 24, 48 and 72 h (Fig. 5c) displayed Annexin-V-FITC staining in 22.8%, 36% and 34% of promastigotes, respectively. Co-staining with annexin-V-FITC and 7-AAD was observed in 30%, 24% and 25% of the population. There was low staining with 7-AAD; 0.8% to 1.3% after 24 to 72 h. The high percentage of single labelling with annexin-V indicates exposure of PS and early apoptosis. The co-staining and the nearly absent labelling of promastigotes with 7-AAD alone reflects apoptosis in an advanced stage in *L. infantum* promastigotes. The dispersion plot (SSC × FSC) shows that promastigotes treated with alendronate have two populations. The cells with volume preserved (over 60% of the total) were composed of unlabeled promastigotes or those only labelled with annexin-V (Fig. 5c). The second population was comprised mainly of promastigotes co-stained with annexin-V and 7AAD and represents damaged promastigotes.

We included controls comprising Annexin-V-FITC- and 7-AAD-labelled promastigotes of *Leishmania* permeabilized with Triton X-100. The forward and side scatter plot (Fig. 5d) demonstrated a loss of membrane integrity and cellular volume. As expected, co-staining with annexin-V-FITC and 7-AAD was observed in 66–74% of promastigotes (Fig. 5d).

### Evaluation of mitochondria membrane potential damage in *Leishmania* treated with N-BP

**Fig. 6:**
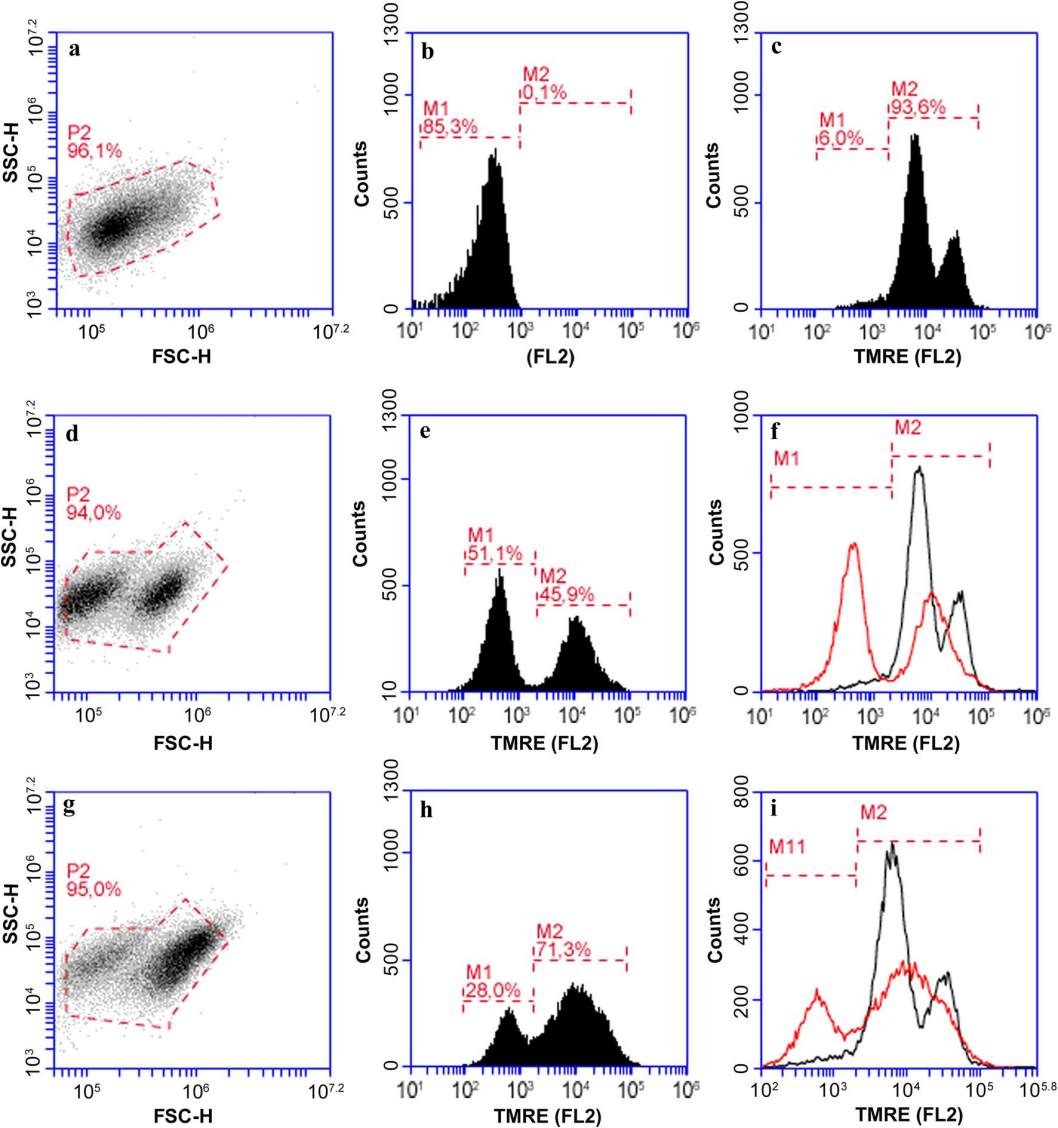
Analysis of mitochondrial membrane potential in promastigotes of *L. infantum* treated with alendronate. Negative control: promastigotes not treated with drugs and not stained with TMRE: light scattering plot shows one population (**a**) with low fluorescence intensity (M1) (**b**). Positive control, promastigotes not treated and stained with TMRE possesses high fluorescence (M2 peaks) (**c**). Treated with 10 µM miltefosine for 72 h and stained with TMRE: light scattering plot shows two populations (**d**) with decreased M2 coupled to an increase in the M1 population (**e**). Treated with 100 µM alendronate for 72 h and stained with TMRE: light scattering plot shows two populations (**g**) with; slight decrease in M2 population to M1 region (**h**). Overlay of positive control (black histogram) with drug-treated parasites (red histogram) (**f**, **i**)

We also evaluated the mitochondrial potential with tetramethylrhodamine ethylesterpercholate (TMRE) in the same culture of *L. infantum* promastigotes treated for 72 h with N-BPs or miltefosine. TMRE is a cationic lipophilic dye that accumulates in the active mitochondrial of viable protozoans; the fluorescent intensity is a direct measure of its accumulation and cellular metabolism. We evaluated *L. infantum* promastigotes untreated with drugs and unlabeled with TMRE, as control, by means of a light scattering plot (SSC × FSC) (Fig. 6a) and FL2 (Fig. 6b). The evaluation of mitochondrial function with TMRE (FL2) displayed that 93.6% of the promastigotes incorporated TMRE, represented by M2. The M2 population displayed two contiguous peaks: one had higher TMRE incorporation and was represented by 25.5% of the promastigotes; and the second one is composed of 68.5% (Fig. 6c). This second group is likely promastigotes in stationary phase, they had reduced division and mitochondrial activity. Similarly, two populations of promastigotes from *L. donovani* were observed with TMRE and increased after 6 and 7 days of cultivation [38].

After incubation with miltefosine and alendronate, another population with low or absent TMRE incorporation appeared and was represented by M1. This population increased with miltefosine concentration from 5 to 10 µM (not shown). The dispersion plot (SSC × FSC) demonstrated two populations of promastigote (Fig 6d, g) indicative of promastigotes with reduced cellular volume and membrane damage. After incubation with TMRE, M1 represented 51.1% of promastigotes affected by 10 µM miltefosine under apoptosis or necrosis, and M2 represented promastigotes with functional mitochondria (45.9%; Fig. 6e). The overlay (Fig. 6f) of the TMRE positive control (Fig. 6c) *versus* promastigotes treated with miltefosine (Fig. 6e) showed a decrease of the M2 subpopulation.

Alendronate (100 µM) abolished mitochondrial membrane potential but in a lower percentage of promastigotes than miltefosine. In our assays, 100 µM alendronate could reduce mitochondrial membrane potential in approximately 28% of promastigotes as represented by M1 (Fig. 6h). The M2 population predominated in promastigotes treated with alendronate (71.3%). An overlay of the histograms shows that the positive control with TMRE and treated with alendronate for 72 h had a M1 population with slight displacement of M2 to the lower fluorescent emission indicating lower TMRE labeling (Fig 6i). As expected, the same population labelled with annexin-V and 7-AAD had a loss of mitochondrial functions.

## Discussion

A previous report compared trypanosomatids with apicomplexans by multiple sequence alignment and predicted seven conserved domains in the FPPS enzyme [19]. We highlighted these conserved domains in the alignment of *Leishmania* FPPS with different *Giardia* assemblages, and FPPS sequences from other organisms (Fig. 1a, c). The *Giardia* FPPS enzyme possesses 405 amino acids, which is about 40 amino acids longer than FPPS from *Leishmania* and trypanosomatids: this is reflected in long internal loops and the carboxyl terminal region (Fig. 1e). The human FPPS carboxy terminal tail has a conserved domain VII and adopts a rigid configuration. The conserved Arg^351^ side chain forms a bridge with the terminal Lys^353^(K). There are amino acid residues that switch on and off the tail configuration [39]. *Leishmania major* and *T. cruzi* FPPS lack this mechanism. However, *Giardia* has 8-mers increase in the carboxyl terminal tail with a possible similar role.

Despite the differences between *Leishmania* and *Giardia* FPPS sequences, the main FPPS signatures were identified in *Giardia* sequences including the FARM motif. This motif allows the classification of FPPS in type I enzymes (eukaryotic origin) or type II enzymes (prokaryotic origin). Type I FARM has a DDXXD signature found in trypanosomatids, *S. cerevisiae*, *H. sapiens and in Giardia* (Fig. 1b), type II displays two extra amino acids inside the FARM sequence, i.e. DDXXXXD sequence [40, 41], as demonstrated in *E. coli* (Fig. 1a). Previous studies demonstrated that aromatic or bulky amino acids at the fourth to fifth amino acids upstream of the FARM motif are fundamental for product specificity and length. They are another characteristic of type I enzymes.

Figure 1a shows *E. coli* FPPS, a type II enzyme without these residues before the FARM motif. These residues are also involved in the binding of NB-P inhibitors and interactions with the phenyl ring of bisphosphonates [41]. The aspartate-rich domains have a role in substrate binding through the phosphates in coordination with Mg^+2^ ions. The carboxyl group of aspartate, residues 98–102 of FARM, and residues 250–254 of SARM coordinate with the phosphate atoms inside the catalytic pocket. The phosphates of N-BP also make hydrogen bonds with the amino groups in Lys^207^ and Lys^264^ [20, 35].

Human FPPS has two phenylalanine residues (Fig. 1a) at the fifth and fourth positions upstream of the FARM motif [20]. In *L. major*, His^93^(H) replaces the phenylalanine at the fifth position, and there is a Phe^94^ at the fourth position. In *T. cruzi* FPPS, both phenylalanines are replaced, the fifth one by His^93^(H), and the fourth by the aromatic amino acid Tyr^94^(Y) [20, 21]. In *Giardia*, the fifth phenylalanine upstream the FARM motif is replaced by Ala^115^(A), but the Phe^116^(F) at the fourth position is maintained. Phenylalanine and tyrosine can limit prenyl chain elongation. Amino acid residues with a smaller side chain (alanine or glycine) can impact the product specificity and increase the space inside the pocket to accommodate longer length prenyl chains. Thus, functional characterization of *Giardia* FPPS, i.e. cloning, mutagenesis and kinetic analysis, can elucidate the amino acids that are involved in the affinity for the substrates, for inhibitors (NB-P), and the prenyl chain product.

Previous work demonstrated that FPPS from *Giardia* formed a separate branch distinct from trypanosomatids (kinetoplastids) and prokaryotes [19], but phylogenetic analysis and multi alignment comparing *Giardia* assemblages with *Leishmania* FPPS proteins were not performed. Thus, we constructed a phylogenetic tree of FPPS enzymes from 17 organisms (Fig. 1f): *Giardia* and *Leishmania* FPPS sequences were placed in distinct clades.

Molecular modelling of *Giardia* FPPS using a threading approach was performed due to the low identity of FPPS from *Giardia* with FPPS from *Leishmania* and other organisms. The model generated from the enzymes helps to explain the conformation adopted by the *Giardia* FPPS including the position of the amino acid residues inside and surrounding the FARM motif and the SARM motif (Fig. 2). These are involved in the binding of the substrate and the inhibitors.

Some studies have tested the activity of N-BPs in different protozoans: *in vitro* assays of *T. cruzi* and *L. donovani* amastigotes inside infected Vero cells, *T. brucei* trypomastigotes, *Toxoplasma gondii* tachyzoites, and *Plasmodium falciparum* intraerythrocytic stages. All models demonstrated that N-BPs, especially aromatic compounds such as risedronate, have significant anti-protozoal activity with an IC_50_ in the nanomolar or low micromolar range [42]. The IC_50_ for N-BPs for intracellular amastigotes of *T. cruzi* was 147 ± 31.2 µM for alendronate and 123 ± 26.4 µM for risedronate. In amastigotes of *L. donovani*, IC_50_ for N-BPs was 82.5 ± 14.6 µM for alendronate and 2.3 ± 0.3 µM for risedronate. This demonstrates that the aromatic N-BP, risedronate, has a better activity in *L. donovani* amastigotes.

Promastigotes of *Leishmania* spp. are good models to evaluate inhibition of the sterol pathway including mechanisms of cell death. They can be used to evaluate the damage and impact on the parasite ultrastructure due to N-BPs and other inhibitors. Despite being the form found in the insect, the dividing promastigotes are easy to cultivate, have active mitochondria, and display high expression of FPPS as evaluated by mRNA and polycistronic RNA [21]. Our study used promastigotes of *L. infantum* and showed that risedronate had higher activity (13.8 ± 6.0 µM) followed by ibandronate (85.1 ± 26.5 µM) and alendronate (112.2 ± 61.2 µM) (Table 2), without toxicity to the host cells as evaluated in RAW and LLCMK2 cells (data not shown).

N-BPs are strong inhibitors of the recombinant FPPS enzyme. N-BPs such as risedronate, alendronate, and pamidronate are competitive inhibitors of IPP and GPP, substrates of FPPS. The recombinant FPPS of *T. cruzi* displayed a higher affinity for risedronate with a *K*_*i*_ of 0.032 µM than alendronate and pamidronate (*K*_*i*_ of 1.04 µM and 2.02 µM, respectively) [22] as estimated by a Dixon plot. Furthermore, when the affinity for each N-BPs was evaluated by the IC_50_, human recombinant FPPS displayed higher affinity for risedronate with an IC_50_ of 0.010 µM *versus T. cruzi* recombinant FPPS with an IC_50_ of 0.037 µM for risedronate [22]. *Leishmania major* recombinant FPPS enzyme also showed higher activities for risedronate (IC_50_ of 0.17 µM) than ibandronate (IC_50_ of 0.48 µM) [21]. These differences in the IC_50_ of the FPPS recombinant enzymes from different organisms can be associated with the amino acid residues found in the binding pocket surrounding the FARM and SARM motifs; these can alter the affinity for the N-BP inhibitors.

Our electron microscopic data showed that N-BPs causes several alterations in intracellular membrane and organelles of *Leishmania* such as myelin figures, mitochondrial swelling, plasma membrane blebs and membrane disorganization (Fig. 3). Previous studies demonstrated that *L. amazonensis* treated with specific inhibitors of ergosterol biosynthesis display morphological alterations and cell death associated with sterol depletion [2]. We also observed Golgi disorganization with small vesicles distributed in the cytoplasm as well as invagination near the flagellar pocket (Fig 3); these are suggestive of alterations in the exocytosis and endocytosis. The inhibition of protein prenylation by bisphosphonates, inhibitors of prenyl protein transferases, or inhibitors of mevalonate or isopentenyl pyrophosphate synthesis (lovastatin, mevastatin and phenylacetate) can profoundly affect cell morphology, cell replication, intracellular signal transduction, and lead to cell death by apoptosis, as demonstrated elsewhere [22, 24].

Programmed cell death was described previously in trypanosomatids and in *L. donovani* promastigotes and amastigotes, caused by parasites in stationary phase or induced by pentostan and amphotericin B. These drugs can induce PPL-cleavage activity, change membrane integrity, increase the electron density in the cytoplasm, and lead to nuclear condensation [38]. Previous publications describing *T. cruzi* epimastigotes treated with the sterol biosynthesis inhibitors, ketoconazole and lovastatin, indicated branching of the mitochondrial membranes including a concentric pattern of the inner mitochondrial membrane in contact with kinetoplast and myelin figures suggestive of autophagy [43].

There are also reports of increased intensity in the membrane potential after rhodamine treatment [43]. In contrast, we did not observe increased mitochondrial potential, as evaluated *via* TMRE nor branching of mitochondrial membranes in promastigotes by electron microscopy after treatment with N-BP.

Our results suggest that necrosis is not the main mechanism of *L. infantum* promastigotes death caused by N-BPs, only ~ 1% of the promastigotes were stained with 7-AAD alone after 48 to 72 h incubation with alendronate (Fig. 5). Indeed, staining with Annexin-V alone and co-staining with 7-AAD led to 34% and 25% labelling, respectively (Fig. 5), and the co-stained population had a loss of membrane potential (TMRE) (Fig. 6). In contrast, *T. cruzi* epimastigotes treated with ketoconazole and lovastatin for 12 h had marked co-staining with Annexin-V and PI or with PI alone, and very little Annexin-V staining [43].

Instead, we suggest that apoptosis is the main mechanism of death caused by N-BPs in *L. infantum* promastigotes. It was described that inhibition of protein prenylation by N-BP is one of the main mechanisms underlying decreased bone resorption by osteoclasts. Apoptosis has been reported for osteoclasts treated with N-BPs [44]. Indeed, N-BPs such as clodronate, etidronate, pamidronate, alendronate, and risedronate in concentrations of 10 and 1000 µM induced apoptosis in Caco-2 human epithelial cells [45]. A recent *in vivo* study demonstrated that subcutaneous administration of zoledronic acid in mice inhibits prenylation of Rab1A, Rab5B, Rab7A, and Rab14 in mouse peritoneal macrophages [46].

Ubiquinone or coenzyme Q (CoQ), a component of the electron transport chain in aerobic organisms as *Leishmania*, can have biosynthesis affected by inhibition of FPPS due to N-BPs, which can be correlated also with the mitochondrial damage observed by electron microscopy in the promastigotes of *L. infantum* treated with risedronate and alendronate. Depending on the *Leishmania* species and life stage, coenzyme CoQ8 and CoQ10 were detected in lower amounts. CoQ9 is the predominant homologue, and it has been detected in all organisms including ones without identifiable mitochondria such as *Giardia*, which has only a reminiscent mitochondrion [15].

The N-BPs activity was more pronounced in *L. infantum* than in trophozoites of *G. duodenalis*, as evaluated by the viability method. We found an IC_50_ of 271 ± 62 µM for ibandronate and 311 ± 120 µM for risedronate (Table 2). The higher activity, anti-proliferative effect of N-BPs, such as risedronate on *L. infantum*, compared to *Giardia*, can relate to the fact that *Giardia* has a minimal sterol metabolism, and to the differences in the catalytic site and pocket of the FPPS in each organism.

The inhibitory concentration of risedronate and ibandronate N-BP was higher in *Giardia* trophozoites than *Entamoeba* indicating a lower activity *versus Entamoeba*. Previous studies evaluated the N-BP activity in amitochondriate *Entamoeba histolytica*, compared to the apicomplexan parasite *Plasmodium*. Trophozoites of *E. histolytica* and *P. falciparum* in the intraerythrocytic stage displayed an IC_50_ above 200 µM for alendronate and pamidronate [47]. The IC_50_ values were 73.5 µM and 123 µM for risedronate and 53.6 µM and 50.1 µM for ibandronate, respectively [47]. In *Giardia* and *E. histolytica*, inhibition of FPPS by N-BP can impact the biosynthesis of dolichol and isoprenoids because ergosterol biosynthesis is absent.

Another aspect to consider is the expression of FPPS in different subcellular compartments, affecting the N-BP intracellular distribution. An FPPS fusion with GFP demonstrated localization in peroxisomes in the amoeba *Dictyostelium discoideum* [48]. An enzyme in mevalonate pathway, 3-hydroxy-3-methylglutaryl-coenyzme A (HMG-CoA) reductase, is an integral enzyme of endoplasmic reticulum (ER) in the amitochondriate *Giardia*. The ER membranes are the site of polyisoprenoids and dolichol biosynthesis.

Trophozoites of *G. duodenalis* treated with N-BPs displayed many more myelin figures (Fig. 4) than *L. infantum* promastigotes. Concentric membranes around the nucleus and around other organelles are indicative of autophagy. The main pathways affected by inhibition of FPPS are the prenylation of proteins and dolichols biosynthesis. In accordance with previous biochemical evidence involving the incorporation of labeled FPP and GGPP isoprenoids in GTP binding proteins, *Giardia* performs isoprenylation of 50 and 21–26 kDa proteins [49]. Prenylation is essential for GTP-binding proteins function, because it is required for protein association to intracellular membranes and for protein-protein interactions including intracellular vesicular transport, membrane endocytosis, and exocytosis.

## Conclusions

Inhibition of the enzyme FPPS by N-BPs can cause a shortage of GPP, FPP and GGPP, which are intermediate metabolites involved in the regulation of cellular functions and homeostasis. A shortage of FPP can cause failure in the isoprenylation of proteins as well as the nuclear lamina and Rab GTPases that are anchored in the intracellular region of the plasma membrane. The nuclear lamina and Rab GTPases interfere with the vesicular transport, endocytosis and exocytosis. Deficits in the synthesis of dolichol interfere with asparagine (N)-linked glycosylation that regulates numerous cellular activities such as glycoprotein quality control, intracellular trafficking and cell-cell communications. These alterations concur with our findings; disorganization of intracellular membranes culminating in *Leishmania* apoptosis. The inhibition caused by NB-P in promastigotes of *Leishmania* and on trophozoites of *Giardia* suggests that they are good models to evaluate protein prenylation and mechanisms of cell death. FPPS is in a branching point in sterol metabolic pathways. It is a key enzyme in the mevalonate pathway and a good candidate for drug design. Based on the catalytic site and mechanism of catalysis of the FPPS in each organism, it is possible to develop specific bisphosphonate inhibitors with high affinity for FPPS expressed in each protozoan.

## Data Availability

Data supporting the conclusions of this article are included within the article.

## Abbreviations

FPPS: farnesyl pyrophosphate synthase
GGPPS: geranylgeranyl diphosphate synthase
N-BP: nitrogen-containing bisphosphonate
IPP: isopentenyl diphosphate
DMAPP: dimethylallyl diphosphate
FPP: farnesyl diphosphate
GGPP: geranylgeranyl diphosphate
NCBI: National Center for Biotechnology Information
BLAST: Basic Local Alignment Search Tool
PDB: protein data bank
MAFFT: multiple alignment using fast Fourier transform
MTS: tetrazolium 3-(4,5-dimethyl-2-thiazolyl)-5-(3-carboxymethoxyphenyl)-2-(4-sulfophenyl)-2H
PMS: 5-methyl-phenazinium methyl sulfate
7-AAD: 7-amino-actinomycin
TMRE: tetramethylrhodamine ethyl ester
SBG: 21 mM Hepes, 0.7 mM Na_2_PO_4_, 137 mM NaCl, 5 mM KCl, pH 7.4, supplemented with 6 mM glucose
